# Genetic variation in transgenerational immune priming and its association with fecundity and body mass in the mealworm beetle *Tenebrio molitor*

**DOI:** 10.1038/s41437-026-00850-9

**Published:** 2026-05-28

**Authors:** Julien Dhinaut, Manon Chogne, Camille Sabarly, Aude Balourdet, Charlène Develay, Maria Teixeira, Nelly Debrosse, Agathe Crosland, Thierry Rigaud, Yannick Moret

**Affiliations:** Biogéosciences, UMR 6282, CNRS Université Bourgogne Europe, Dijon, France

**Keywords:** Evolutionary ecology, Structural variation, Evolutionary biology

## Abstract

Understanding how organisms allocate resources between self-maintenance and reproduction in response to infection is central to life-history evolution. Maternal transfer of immune protection to offspring, often referred to as transgenerational immune priming (TGIP), represents a potentially important component of reproductive allocation. However, the extent to which this maternal effect varies genetically and covaries with other life-history traits remains poorly understood. Here, we investigated genetic variation in maternal transfer of immune protection and its association with key life-history traits in the mealworm beetle *Tenebrio molitor*. Using ten inbred lines, we quantified maternal body mass, fecundity, investment in offspring immune protection, and survival under starvation following a bacterial immune challenge. This design allowed us to estimate the broad-sense heritability of maternal immune transfer in an invertebrate and to assess genetic correlations with other traits. We found significant genetic variation in maternal immune transfer, fecundity, and body mass. Investment in offspring immune protection was positively correlated with fecundity but negatively correlated with body mass, while its association with survival was negative but not statistically significant. Smaller females provided more immune protection to their offspring but produced fewer offspring, suggesting a potential trade-off between reproductive effort and offspring immunity depending on body size. Overall, our results suggest that maternal transfer of immune protection in *T. molitor* is an evolvable trait that is integrated with broader life-history variation. More broadly, this study highlights the importance of considering maternal immune effects as an integrated component of reproductive allocation, potentially shaping adaptive immune investment within species.

## Introduction

Upon infection, hosts face trade-offs between investment in immune defence and other life history traits such as reproduction. Allocating resources to immunity can reduce those available for reproduction, while prioritizing reproduction may compromise the ability to cope with infection (Schwenke et al. 2016). These trade-offs are central to life-history theory and contribute to variation in reproductive allocation among individuals (Williams 1966; Roff 1992; Stearns 1992).

Because both the number and quality of offspring contribute to fitness, resource allocation to reproduction may involve a trade-off not only between reproduction and other functions, but also between offspring number and offspring quality. In environments where parents and offspring face similar threats, such as parasites, producing offspring with high immune defences can enhance offspring survival.

One way mothers achieve this is through the transfer of immune protection to their offspring, whereby maternal immune experience influences offspring immunity, preparing them for prevalent pathogens likely to be encountered (Grindstaff et al. 2003; Hasselquist and Nilsson 2009; Tetreau et al. 2019). Maternal transfer of immunity is well documented in vertebrates, where infected mothers pass antibodies to their offspring for temporary protection (Grindstaff et al. 2003; Hasselquist and Nilsson 2009). Maternal transfer of immunity also occurs in invertebrates, though it involves immune proteins rather than antibodies (Tetreau et al. 2019; Vilcinskas 2021). In the mealworm beetle *Tenebrio molitor*, for example, microbially infected females transfer antimicrobial compounds to their eggs (Dubuffet et al. 2015; Tetreau et al. 2020). The developing offspring exhibit improved survival upon infection, even after reaching the adult stage (Dhinaut et al. 2018a; Zanchi et al. 2011). This process is commonly referred to as transgenerational immune priming (TGIP) in invertebrates (Moret 2006).

However, transferring immune protection to offspring is not without costs. In *T. molitor*, the transfer of antibacterial compounds to eggs reduces the mother’s own immune response (Moreau et al. 2012) and decreases her fecundity (Zanchi et al. 2012). Costs may also occur in offspring, as maternal immune transfer has been associated with prolonged larval development in several beetle species (Roth et al. 2010; Zanchi et al. 2011; Dhinaut et al. 2018a; Schulz et al. 2019), reduced reproductive performance in moths (Trauer and Hilker 2013), and decreased resistance to novel parasites in bumblebees (Sadd and Schmid-Hempel 2009).

The evolutionary factors shaping variation in maternal immune transfer remain poorly understood, particularly in relation to other life-history traits. For instance, the insect immune response is known to be genetically variable (Rolff et al. 2005; Hammerschmidt et al. 2012; Letendre et al. 2021), and such variation may help explain why immune transfer to offspring also varies among invertebrate populations (Khan et al. 2016). This suggests a heritable basis, and because only heritable traits can respond to natural selection, assessing the genetic basis of maternal immune transfer is essential to understanding its evolution. However, to our knowledge, no formal investigation of genetic variation in maternal immune transfer has been conducted in invertebrates. In addition, how this maternal trait is genetically integrated with other life-history traits remains poorly understood.

Comparative studies suggest that this form of immune investment is more common in long-lived invertebrates than in short-lived ones (Pigeault et al. 2016). This pattern is consistent with predictions from the pace-of-life syndrome (Réale et al. 2010), which links life history traits such as lifespan, development rate, and reproductive allocation with investment in individual immunity (Martin et al. 2006, 2007; Sparkman and Palacios 2009; Niemelä et al. 2013). However, while this framework has been commonly used to explain variation in individual immune investment (Niemelä et al. 2013), its relevance for maternal investment in offspring immunity remains unclear. One might predict that slow-living females, who invest more in individual immunity, may survive infections and delay reproduction, possibly investing less in their offspring's immunity to conserve resources for future reproductive opportunities. On the other hand, fast-living females, facing low survival probabilities due to infection, might benefit from increased investment in protected offspring during early reproduction, even at the expense of their own immunity and survival (Moreau et al. 2012). However, these complementary predictions to the pace-of-life syndrome hypothesis remain to be explored.

Here, we investigated variation in maternal transfer of immunity using the mealworm beetle *Tenebrio molitor* as a model. This beetle is a cosmopolitan pest of stored grain products and is currently raising considerable interest as an alternative protein source for food and feed (Vigneron et al. 2019). Females begin reproducing from their fifth day post-eclosion, can mate many times with different males, and exhibit peak fertility between 10 and 20 days of age, which then gradually declines until death a few weeks later (Jehan et al. 2020). Previous work showed that immune challenges in early or late-life reduce fecundity without affecting female survival (Jehan et al. 2022). Following a bacterial immune challenge, females transfer antimicrobial activity to their eggs, whereas non-immune-challenged females either do not provide such protection or do so only rarely (Dubuffet et al. 2015; Tetreau et al. 2020). This maternal transfer is transient and varies in the proportion of protected eggs among immune-challenged females (Zanchi et al. 2012). The level of protection per egg has been shown to be traded off against the mother’s own antibacterial immune response, but this trade-off appears to occur only in small females (Moreau et al. 2012). Body mass, used as a proxy for individual quality, may therefore play an important role in the expression of maternal immune transfer, as it correlates positively with energetic reserves (Zanchi et al. 2022) and resistance to infections (Monceau et al. 2017; Jehan et al. 2021). Furthermore, body mass is linked to development speed: individuals who develop quickly tend to be smaller than those who develop more slowly (Crosland et al. 2022, 2024). These life-history dynamics are not only relevant in natural populations but also under artificial selection pressures. For example, farming practices in *T. molitor* often favour rapid development and high reproductive output. While such conditions may inadvertently select against high investment in individual immunity, their implications for the evolution of maternal immune transfer remain unclear. Investigating how maternal transfer of immunity correlates with other life-history traits in *T. molitor* can therefore provide valuable insights for insect farming, particularly given that mass-rearing conditions often carry a high risk of disease transmission and outbreaks (Vigneron et al. 2019).

In this study, we investigated phenotypic and genetic variation in early-life maternal immune transfer, body mass, fecundity, and survival under starvation following a mimicked pathogenic challenge in *T. molitor*. Using inbred lines, we assessed the heritability of maternal immune transfer and examined its genetic correlations with key life-history traits. This approach allowed us to examine how maternal immune investment relates to variation in life-history traits.

## Materials and methods

### Beetle cultures

*Tenebrio molitor* used in this study originated from an outbred stock culture of more than 100,000 individuals, maintained under standard laboratory conditions (24 ± 2 °C, 70% RH; permanent darkness) and allowed to breed randomly. Ten inbred lines (designated A to J) were created by subjecting a random subset of beetles from this population to five generations of full-sib mating (only one inbred pair per generation), followed by free mating in panmixis among the offspring within each line. Each inbred line was then maintained in two 47 L containers, each hosting several hundred individuals. Individuals from both containers were mixed every generation to maintain genetic unity while introducing environmental variation. All the experimental beetles were reared and kept in an insectary at 24 ± 2 °C, 70% relative humidity, under constant darkness, and were supplied *ad libitum* with bran flour, water, and apple as a supplement. Beetles used in this study were virgin adults of controlled age (10 ± 2 days post-eclosion), randomly sampled as pupae from both containers from each inbred line or the outbred stock culture. Before being used in the study, all beetles were weighed to the nearest 1 mg using an OHAUS balance (Discovery Series, DU114C).

### Experimental design

Groups of 17 females per inbred line were used to characterize maternal investment in TGIP following a standard maternal immune challenge. TGIP was analysed as a function of female body mass, fecundity, and post-reproductive survival. The data allowed us to quantify genetic variation among beetle lines for these maternal traits by calculating their respective heritabilities and testing for correlations among them. Since our focus was on genetic variation in maternal investment in TGIP across the beetle lines, all females were immune-challenged to induce TGIP, as it is a prerequisite for its expression. Indeed, there is no maternal transfer of immunity if females are not immune-challenged (Zanchi et al. 2012; Dubuffet et al. 2015; Dhinaut et al. 2018b). Consequently, for the purpose of our study, control females (either injected with saline solution or unmanipulated) were not necessary.

Using inbred organisms in evolutionary research can be problematic, particularly when inbreeding depression leads to a significant reduction in fitness or fitness-related traits, or when the inbred lines do not accurately represent a random sample of genotypes from the original outbred population (Archer et al. 2012). Conversely, purging of recessive deleterious alleles during inbreeding may further bias the surviving lines toward genotypes with higher fitness, potentially altering trait distributions and reducing genetic variance. Inbreeding can also distort quantitative genetic parameters, especially the direction and magnitude of genetic correlations between traits (Rose 1984). Therefore, to illustrate the potential impact of inbreeding on trait expression, we included 17 females from the outbred population, alongside the inbred lines, and measured their investment in TGIP following a standard maternal immune challenge, as well as body mass, fecundity, and post-reproductive survival, as outlined above.

While we did not specifically aim to test for inbreeding effects, we compared traits between outbred and inbred lines (see below) to establish a baseline for comparison. The females from the outbred population were not included in further statistical analyses but were incorporated into the figures for visual comparison, helping determine whether the trait means for the inbred lines fell within the natural range observed in the outbred population. However, there may still be effects of inbreeding on quantitative genetic estimates. Therefore, as with any study involving inbred lines, caution is needed when interpreting our quantitative genetic estimates.

Maternal investment in offspring immune protection, following a standard benign bacterial immune challenge, was estimated based on the proportion of eggs that show antibacterial activity and the level of antibacterial activity in protected eggs (Zanchi et al. 2012). To conduct the immune challenge, we used the gram-positive bacterium *Bacillus thuringiensis*, known to be a common bacterial pathogen of coleopteran insects (Jurat-Fuentes and Jackson 2012). For each inbred line, 17 virgin adult females were weighed to the nearest mg and underwent an immune challenge by being injected with a 5 µL suspension of inactivated *B. thuringiensis* in sterile phosphate-buffered saline (PBS, 10 mM, pH 7.4) after being chilled on ice for 10 minutes (Zanchi et al. 2012). Inactivation of the bacteria was done by fixation in formaldehyde solution (see below for the method). Following the immune challenge, the females were paired with a virgin and immunologically naive male from the outbred stock culture. They were then allowed to produce eggs for 8 days after the immune challenge in Petri dishes supplied with wheat flour, apple, and water under standard laboratory conditions. Similarly, 17 virgin adult females from the outbred stock culture were used as a control group to compare with the inbred lines. The total number of eggs produced by each female during the 8 days following their immune challenge was recorded. However, only the eggs produced between days 2 and 8 after the maternal immune challenge were used to test their antibacterial activity, as previous studies have shown that immune-challenged females of *T. molitor* mainly protect their eggs within this period (Zanchi et al. 2012). Furthermore, it was found that the eggs of females immune-challenged with *B. thuringiensis* require 3 days of development after being laid to exhibit antibacterial activity (Dhinaut et al. 2018b). As a result, the eggs were kept under standard laboratory conditions for 3 days post-oviposition before being frozen in liquid nitrogen and stored at –80 °C, pending measurement of their antibacterial activity. On the 8th day after their immune challenge, females were isolated in grid boxes (boxes with 10 compartments; each compartment: L x W x H, 4.8 ×3.2 ×2.2 cm) without food. The females were checked once a week for survival under starvation to assess their remaining resources for survival (Moret and Schmid-Hempel 2000). Therefore, this approach allowed us to estimate female body mass, fecundity within 8 days post-challenge, investment in egg protection, and survival under starvation after reproduction.

### Bacterial cultures for immune challenges

Bacterial cultures and immune challenges were performed as described by Dhinaut et al. (2018b). The bacterium *B. thuringiensis* (CIP53.1) used for the immune challenges was obtained from the Pasteur Institute. The bacteria were grown overnight at 28 °C in liquid Broth medium (10 g bacto-tryptone, 5 g yeast extract, and 10 g NaCl in 1000 mL of distilled water, pH 7). Afterward, the bacteria were inactivated for 30 minutes in 0.5% formaldehyde prepared in PBS, rinsed three times in PBS, and their concentration was adjusted to 10^8^ bacteria per mL using a Neubauer improved cell counting chamber under a phase-contrast microscope (magnification x 400). The success of the inactivation was verified by plating a sample of the bacterial solution on sterile Broth medium with 1% bacterial agar and incubating it at 28 °C for 24 hours. Aliquots of the bacterial suspension were kept at −20 °C until use. Immune challenges of the beetles were performed by injection through the pleural membrane between the second and third abdominal tergites using sterile glass capillaries that had been pulled out to a fine point with an electrode puller (Narashige PC-10).

### Egg antibacterial activity

The antimicrobial activity of each collected egg was measured using a standard zone-of-inhibition assay (Dhinaut et al. 2018b). Individual egg samples were thawed on ice, and egg extracts were prepared by homogenizing each egg into an acetic acid solution (0.05%, 5 μL per egg). After centrifugation (3500 g, 2 min, 4 °C), 2 µL of the supernatant was applied to zone-of-inhibition plates seeded with *Arthrobacter globiformis* (CIP105365), obtained from the Pasteur Institute. *A. globiformis* is commonly used as the target bacterium in antibacterial assays due to its high sensitivity to insect-derived antibacterial compounds, which enhances assay resolution (Dubuffet et al. 2015). This sensitivity is crucial for detecting antibacterial activity at the level of individual eggs. Zone inhibition plates were prepared from an overnight culture of *A. globiformis*, which was added to Broth medium containing 1% agar to reach a final concentration of 10^5^ cells per mL. Six millilitres of this seeded medium were poured into Petri dishes and allowed to solidify. Sample wells were made using a Pasteur pipette fitted with a ball pump. Two microliters of the sample solution were added to individual wells. A positive control (Tetracycline: Sigma-Aldrich, St. Louis, MO, USA, T3383; 2.5 mg/mL in absolute ethanol) was included on each plate (Dhinaut et al. 2018b). Plates were incubated overnight at 28 °C, after which the diameter of each inhibition zone was measured. Due to slight variations in inhibition zones in tetracycline controls, sample inhibition zones were normalized. This was achieved by adjusting each sample’s zone diameter based on the ratio between the tetracycline control on a reference plate (the plate showing the largest inhibition zone) and the tetracycline control on the plate where the sample was measured (Dhinaut et al. 2018a, 2018b).

From this assay, we estimated female investment in egg protection using two complementary measures. First, the proportion of protected eggs per female was calculated as the number of eggs exhibiting antibacterial activity (i.e., showing a detectable zone of inhibition) divided by the total number of eggs produced. Second, among the subset of eggs that showed antibacterial activity, the mean diameter of the inhibition zone was used to estimate the amount of antimicrobial compounds transferred to the eggs. These two measures respectively captured (1) the proportion of offspring receiving protection and (2) the level of protection provided to those offspring.

### Statistics

We compared maternal trait measures between outbred and inbred females with linear mixed-effects models (LMMs) or generalized linear mixed-effects models (GLMMs) implemented in the lme4 package (Bates et al. 2015) in R (R Core Team 2015), including a binary fixed effect for inbreeding status (inbred vs. outbred) and controlling for body mass. All inbred lines were pooled into a single category (‘inbred’), while the outbred population was coded as ‘outbred’. Body mass was included as a covariate, as it is a proxy for female quality in *T. molitor* (Jehan et al. 2020, 2021, 2022; Crosland et al. 2022; Zanchi et al. 2022), and line identity was included as a random factor. The proportion of protected eggs laid by each female was analyzed with a binomial GLMM, taking into account the number of protected eggs and the number of eggs tested per female. Fecundity (total egg number) and survival time under starvation were analyzed using Poisson GLMMs, whereas levels of antibacterial activity in protected eggs were analyzed with an LMM. Finally, we tested the effect of inbreeding on female body mass itself with an LMM that included line identity as a random effect.

Phenotypic relationships between the two measures of maternal investment in egg protection (the proportion of protected eggs among those tested, and the average antibacterial activity of protected eggs from each female) and female body mass, fecundity, and starvation resistance were tested using LMMs or GLMMS as above. Female body mass, fecundity, and starvation resistance were included as covariates, with the inbred line treated as a random factor. The outbred population was not included in this analysis.

To assess the potential heritability of maternal investment in egg protection (measured both as the proportion of protected eggs among those tested and as the average antibacterial activity of protected eggs from each female), body mass, fecundity, and post-reproductive survival, we calculated the repeatability of each trait across lines using the rptR package in R (Nakagawa and Schielzeth 2010; Stoffel et al. 2017). Each of the 10 inbred lines (the original outbred population from which the lines were derived was not included in these analyses) consisted of 17 individuals, and all individuals were measured once for the traits listed above. In this context, repeatability reflects the proportion of phenotypic variance explained by differences among lines and sets an upper bound on broad-sense heritability (*H*²), which in turn represents an upper bound on narrow-sense heritability (*h*²), particularly if additive genetic effects predominate. This approach assumes that lines are genetically homogeneous and that environmental variation is randomly distributed across them. Repeatability was estimated using linear mixed-effects models with “Line” as a random effect and 10,000 bootstrap iterations to compute 95% confidence intervals (CIs). Additionally, we used a likelihood ratio test (LRT), as implemented in the rptR package (REML-based), to assess whether repeatability estimates for each trait were significantly greater than zero. *P*-values from the LRT were adjusted for multiple testing using the Benjamini-Hochberg false discovery rate (FDR) procedure (Benjamini and Hochberg 1995). However, as a conservative criterion, we considered repeatability to be significant only when the 95% CI did not include zero.

Genetic correlations between maternal traits were estimated by accounting for within-line variation for each pair of traits. To this end, we employed a repeated sampling procedure: one female was randomly sampled from each line, and a Pearson correlation coefficient (along with its standard error) was calculated for all pairs of traits. For each correlation estimate, the jackknife resampling method was applied following the approach of Roff and Preziosi (1994). Specifically, a series of N (in this case 10) pseudo values was computed by sequentially excluding each line one by one and re-estimating the correlation using the formula:

$${S}_{N,{i}}=\,{N}_{{rN}}-\,{\left(N-1\right)}_{{rN}-1,{i}}$$

where *S*_*N,i*_ is the *i*^th^ pseudo value, *r*_*N*_ is the correlation coefficient estimated using one randomly sampled female per inbred line across all *N* lines, and *r*_*N−1,i*_ is the correlation coefficient calculated after excluding the *i*th inbred line (Archer et al. 2012; Letendre et al. 2021). The jackknife estimate of the genetic correlation (*r*_*j*_) is the mean of the pseudo values, and an estimate of the standard error (SE) is given by:

$${SE}=\frac{{\sum }_{i=1}^{i=N}{\left({S}_{N,i}-{r}_{j}\right)}^{2}}{N\left(N-1\right)}$$

The entire procedure, comprising random sampling of one female per line, correlation estimation, jackknife resampling, and computation of *r*_*j*_ and SE, was repeated 10,000 times. From these iterations, we calculated the mean genetic correlation coefficient, its average standard error, and the proportion of iterations in which the correlation was statistically significant.

According to simulation models, the jackknife method yields more precise genetic estimates than traditional inbred line means when fewer than 20 inbred lines are used (Roff and Preziosi 1994). These genetic (co)variance estimates from inbred lines include dominance or epistasis variance and are thus considered broad-sense estimates (Falconer and Mackay 1996). By rearing beetles individually, we reduced the variance between lines caused by shared environmental factors and interactions (Archer et al. 2012; Letendre et al. 2021). Average genetic correlations were considered statistically significant if their ratios to average standard errors exceeded 1.96, rejecting the null hypothesis of no correlation based on a two-tailed t-distribution with infinite degrees of freedom.

## Results

### The effects of inbreeding on TGIP, fecundity, body mass, survival under starvation, and body mass

**Fig. 1:**
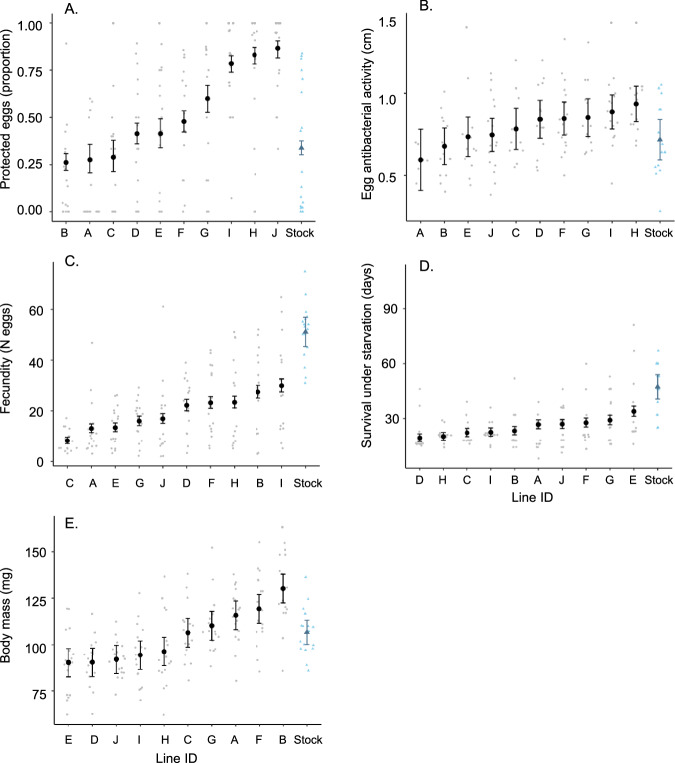
Variation in transgenerational immune priming and life-history traits among inbredlines. Variation (marginal mean ± 95% confidence interval) in **A** the proportion of protected eggs, **B** antibacterial activity of protected eggs, **C** maternal fecundity, **D** survival under starvation, and **E** body mass among 10 inbred lines (lines A to J, filled black circles) derived from the laboratory outbred stock population (*Stock*, filled dark blue triangle) of *Tenebrio molitor*. Grey dots represent individual data points, illustrating within-line variation.

Inbreeding had no significant effect on both measures of the expression of immune transfer (Table 1, Fig. 1A, B). However, inbred lines significantly exhibited lower fecundity and post-reproductive survival under starvation (Table 1, Fig. 1C, D). Overall, the proportion of protected eggs was negatively correlated to female body mass (Table 1). Female body mass was also slightly negatively impacted by inbreeding (Table 1, Fig. 1E).

**Table 1:** Results of generalized mixed-effects models examining the effects of inbreeding (inbred *versus* outbred) on maternal transfer of immunity to eggs and maternal traits.

Traits	Model term	*Estimate*	*S.E*.	*z / t*	*p*
Prop. Protected Eggs (*Binomial*)	Inbreeding	0.80	0.96	0.83	0.406
	Body mass	−0.01	0.002	−3.55	<0.001
Egg ABA (*Gaussian*)	Inbreeding	0.69	0.10	0.68	0.519
	Body mass	0.001	0.001	1.17	0.247
Mother fecundity (*Poisson*)	Inbreeding	−0.29	0.11	−2.62	0.009
	Body mass	0.15	0.02	7.88	<0.001
Mother survival (*Poisson*)	Inbreeding	−0.19	0.05	−3.73	<0.001
	Body mass	−0.07	0.02	−3.97	<0.001
Mother body mass (*Gaussian*)	Inbreeding	−2.17	14.74	−0.15	0.022

Response variables include the proportion of protected eggs (Prop. Protected Eggs), the mean level of antibacterial activity in protected eggs (Egg ABA), maternal fecundity, survival under starvation, and female body mass. Female body mass was included as a covariate for all dependent variables except for body mass itself. Line identity was modeled as a random effect. The error distribution used for each model is indicated in parentheses.

### Phenotypic covariance between maternal investment in TGIP, fecundity, body mass, and survival under starvation

**Fig. 2:**
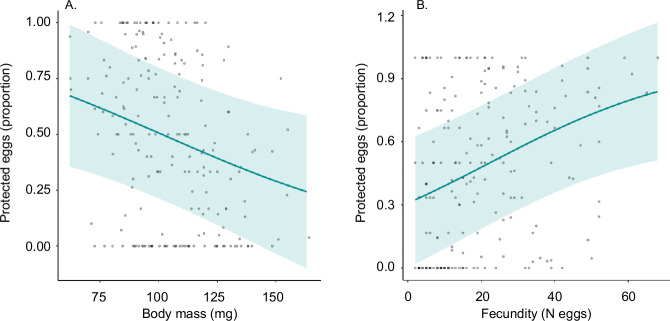
Maternal investment in transgenerational immune priming covaries with female body mass and fecundity. Phenotypic covariation between the proportion of protected eggs and **A** female body mass, and **B** female fecundity among 10 inbred lines of *Tenebrio molitor*. Regression lines represent model predictions, with shaded green areas indicating the standard error (SE) around each line. Grey dots represent individual data points.

Among the inbred lines, the proportion of protected eggs was significantly influenced by both female body mass (Estimate ± SE: -0.018 ± 0.003, χ² = 37.86, df = 1, *P* < 0.001) and fecundity (Estimate ± SE: 0.036 ± 0.004, χ² = 105.50, df = 1, *P* < 0.001). Specifically, heavier females protected a smaller proportion of their eggs (Fig. 2A), whereas females with higher fecundity protected a greater proportion (Fig. 2B). Post-reproductive survival under starvation did not significantly affect the proportion of protected eggs (χ² = 2.73, df = 1, *P* = 0.098). Neither female body mass (*t* = 1.28, *P* = 0.198), fecundity (*t* = 0.77, *P* = 0.446), nor survival (*t* = 0.71, *P* = 0.481) significantly explained variation in antibacterial activity of protected eggs.

### Heritability and genetic covariance between maternal investment in TGIP, fecundity, body mass, and survival under starvation

Phenotypic variation observed among the ten inbred lines (Fig. 1) revealed significant repeatability for the proportion of protected eggs, fecundity, and maternal body mass (Table 2), indicating substantial broad-sense heritability for these traits. Weak *p*-values (0.05 > *p* > 0.01) were considered non-significant when the 95% confidence intervals of the repeatability estimates included zero (Table 2).

**Table 2:** Repeatability estimates used as proxies for broad-sense heritability (*H*²) for maternal investment in immune egg protection (Prop. Protected Eggs: proportion of eggs protected; Egg ABA: antibacterial activity of protected eggs), maternal fecundity, body mass, and survival under starvation, based on data from 17 females per each of 10 inbred lines of *Tenebrio molitor*.

Traits	*Repeatability*	*SE*	*CI 95%*	*Adjusted P-value*
Prop. protected eggs	0.217*	0.090	[0.046 ; 0.395]	<0.001
Egg ABA	*0.103*	0.071	[0 ; 0.261]	0.034
Mother fecundity	0.158*	0.073	[0.012 ; 0.295]	<0.001
Mother body mass	0.411*	0.123	[0.142 ; 0.62]	<0.001
Mother survival	*0.073*	0.049	[0 ; 0.176]	0.019

Estimates with confidence intervals not including zero are considered significant and are marked with an asterisk (*). Estimates shown in italics are considered non-heritable, despite adjusted *P*-values < 0.05, due to low repeatability and confidence intervals that include zero.

**Fig. 3:**
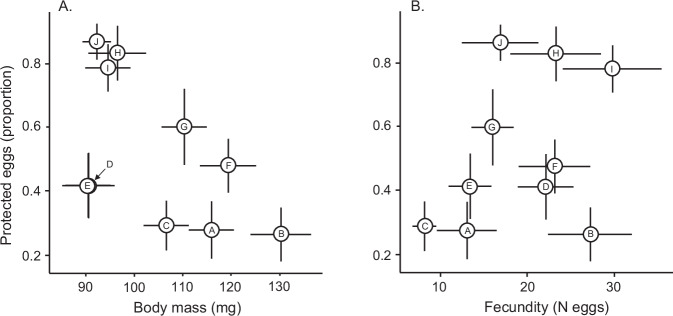
Genetic covariation between maternal investment in transgenerational immune priming, female body mass, and fecundity. Genetic covariation between the proportion of protected eggs and **A** female body mass, and **B** female fecundity among the 10 inbred lines of *Tenebrio molitor*. Each point represents the arithmetic mean ± standard error for a given line.

Significant genetic correlations were found between the proportion of eggs protected by females and both body mass and fecundity (Table 3). Specifically, the proportion of protected eggs was negatively correlated with female body mass (Fig. 3A), but positively correlated with fecundity (Fig. 3B). No other significant correlations were detected (Table 3).

**Table 3:** Matrix of average estimated genetic correlations ( ± average standard errors) among maternal traits: immune egg protection (Prop. Protected Eggs: proportion of eggs protected; Egg ABA: antibacterial activity of protected eggs), maternal fecundity, body mass, and survival.

	Prop. protected eggs	Egg ABA	Fecundity	Mass
Egg ABA	0.20 ± 0.22 (50.5%)			
Fecundity	0.28* ± 0.12 (60.4%)	0.07 ± 0.22 (44.7%)		
Mass	−0.36* ± 0.10 (71.1%)	0.00 ± 0.23 (44.6%)	0.15 ± 0.14 (50.9%)	
Survival	−0.12 ± 0.14 (58.6%)	−0.05 ± 0.23 (44.1%)	0.00 ± 0.12 (49.1%)	−0.08 ± 0.14 (53.1%)

Estimates are based on 10,000 iterations involving random sampling of one female per line, correlation estimation, jackknife resampling, and computation of the correlation coefficient and its standard error (SE). Asterisks indicate statistically significant correlations, defined as those with an estimate-to-SE ratio greater than 1.96. The proportion of iterations in which the correlation was statistically significant is shown in parentheses.

## Discussion

Transgenerational immune priming (TGIP) is an increasingly studied phenomenon in invertebrates, with numerous studies focusing on its taxonomic distribution as well as the physiological and molecular mechanisms underlying its expression (Tetreau et al. 2019; Vilcinskas 2021). However, the evolutionary processes that shape the emergence and maintenance of this maternal effect remain poorly understood. To our knowledge, this study is the first to formally investigate genetic variation in maternal immune transfer in an invertebrate, suggesting a heritable basis for this trait, which may help explain why immune transfer to offspring varies among invertebrate populations (Khan et al. 2016), thereby addressing a key gap in our understanding of its evolutionary potential.

In our study, despite significant phenotypic variation in all measured maternal traits across the ten inbred beetle lines, only maternal investment in egg immune protection (i.e., the proportion of protected eggs), female body mass and fecundity exhibited significant genetic variation (Table 1). Repeatability estimates for these traits, which set the upper limits of heritability, were relatively high, consistent with expectations under laboratory conditions with reduced environmental noise (Roff and Simons 1997). This indicates that these traits, including maternal immune transfer, have the potential to respond to natural selection. If part of this variation is of additive genetic origin, these traits could potentially respond rapidly to natural or artificial selection.

In contrast, the low (and non-significant) repeatability of the other maternal traits could possibly be attributed either to strong directional selection having substantially eroded genetic variation during artificial breeding (Mousseau and Roff 1987; Falconer and Mackay 1996; Merilä and Sheldon 1999), or to large residual variance due to multiple factors influencing trait expression (Wheelwright et al. 2014). Further quantitative genetic studies will be necessary to clarify this issue.

The use of inbred lines derived from a common outbred population in this study provides a valuable, albeit not ideal, framework for estimating broad-sense heritability and genetic correlations for maternal life-history traits. Reduced within-line genetic variation and a controlled environment enhance the reliability of between-line variance estimates, thereby highlighting among-line differences that reflect genetic effects (Lynch and Walsh 1998). However, we also detected evidence of inbreeding depression in fecundity, body mass, and survival under starvation, consistent with the expression of deleterious recessive alleles (Charlesworth and Charlesworth 1999). In contrast, the expression of maternal immune transfer was not affected by inbreeding, suggesting that it may be less sensitive to such genetic load or regulated by more robust genetic or epigenetic mechanisms. This finding strengthens the interpretation of the observed positive genetic and phenotypic correlation between maternal transfer of immunity and fecundity, and the negative correlation with female mass, as these associations are unlikely to result from parallel inbreeding effects. Nevertheless, care should be taken when extrapolating these results to outbred or natural populations, where genetic diversity and environmental interactions are more complex (Hoffmann and Merilä 1999; Kruuk 2004).

Understanding how maternal transfer of immunity covaries genetically with other maternal traits, such as fecundity, body mass, and survival, provides insights into how this maternal effect may contribute to the evolution of female reproductive strategies. This suggests that maternal immune transfer may not evolve independently, but as part of a broader life-history syndrome. Notably, our results show that maternal investment in egg protection was positively correlated with fecundity. Although not statistically significant, we also observed a trend toward a negative correlation between the proportion of protected eggs and survival under starvation conditions after a reproductive episode. While this pattern is consistent with traits associated with terminal investment, whereby individuals allocate more resources to current reproduction at the expense of future survival (Charlesworth and Léon 1976; Clutton-Brock 1984), our results demonstrate genetic covariance rather than direct evidence that maternal immune transfer itself constitutes a terminal investment decision. Accordingly, our study does not directly test plastic reproductive strategies such as terminal investment or reproductive restraint, but instead examines how maternal immune transfer is genetically integrated with life-history traits. While previous work using *T. molitor* showed that early- or late-life immune challenge did not consistently affect fecundity in a way consistent with a terminal investment (Jehan et al. 2022), our results highlight the importance of considering not only offspring number but also offspring quality, such as transgenerational immune protection, when assessing reproductive allocation.

We found that smaller females invested more in egg protection than larger ones, as evidenced by a negative correlation between body mass and maternal immune transfer. In *T. molitor*, large body mass typically reflects higher body condition, longer lifespan, greater later-life reproductive effort and stronger resistance to infection (Monceau et al. 2017; Jehan et al. 2021; Zanchi et al. 2022; Crosland et al. 2022, 2024). However, body mass in this species is also tightly linked to development speed and broader pace-of-life trajectories, with faster-developing individuals tending to be smaller (Crosland et al. 2022). Yet, body mass did not significantly correlate with fecundity or survival under starvation following an immune challenge. One possible explanation is that lighter females increased their resource allocation to reproduction, while heavier females reduced it in response to the immune stimulus, resulting in comparable overall fecundity. Moreover, since the immune challenge used inactivated bacteria that triggered immune responses without causing disease, larger females may have invested more energy in immune resistance, whereas smaller females may have tolerated the infection and prioritized reproduction. If the bacteria had been alive, smaller females might have experienced higher mortality than larger ones. Thus, body-size–dependent differences in immune strategy may explain the absence of a relationship between body mass and survival. Importantly, smaller females should therefore not necessarily be interpreted as “low-quality” individuals per se, but may instead reflect a faster pace-of-life strategy, which aligns with predictions of the pace-of-life syndrome framework.

Importantly, our findings may have implications for the study of immunological priming both within and across generations. Immune priming is viewed as an anticipatory defence system against repeated infections by parasites and pathogens across generations. The risk of re-infection by a parasite or pathogen usually increases with host lifespan. Mathematical models suggest that while short-lived organisms may die before being re-infected, intermediate- and long-lived organisms benefit from investing in individual immune priming because they are more likely to face re-infection (Best et al. 2013). It has been suggested that this could apply to transgenerational immune priming as well, which appears to occur more frequently in long-lived than in short-lived invertebrates (Pigeault et al. 2016). However, our within-species quantitative genetic results indicate that, at least in *T. molitor*, maternal immune transfer covaries with traits characteristic of faster life histories. If, as our study suggests, this maternal effect is associated with traits characteristic of faster life histories within this species, it could imply a trade-off between individual immune priming and transgenerational immune priming. Future studies should test this hypothesis for a better understanding of the evolution of both individual and transgenerational immune priming.

These findings may have practical implications for insect farming, where selection for rapid development and high fecundity often dominates breeding strategies. Under the pace-of-life syndrome hypothesis, such selection may inadvertently reduce investment in individual immunity (Niemelä et al. 2013), increasing vulnerability to disease outbreaks common in mass-rearing systems (Vigneron et al. 2019). However, our results suggest that maternal immune transfer may remain expressed or even be favoured in fast-developing lines, as it is genetically associated with traits characteristic of a fast pace-of-life strategy. Furthermore, we found that the immune protection conferred to offspring was not negatively affected by inbreeding, a factor commonly present in mass-reared insect populations for food and feed. While these effects may partially compensate for a potential decline in individual immunity under selection for fast development, further investigation is required.

In a broader perspective, studying the relationship between transgenerational immune priming and pace-of-life strategies should not be restricted to females, as transgenerational immune priming has also been shown to result from paternal effects in several species (Roth et al. 2010, 2012; Zanchi et al. 2011; Eggert et al. 2014), including vertebrates (Roth et al. 2012). Furthermore, immune-challenged males have also been found to adjust their reproductive investments toward terminal investment. For instance, in *T. molitor*, immune-challenged males increase their investment in sexual signalling (presumably a costly trait) to enhance their attractiveness to females (Sadd et al. 2006; Kivleniece et al. 2010; Krams et al. 2011, 2015; Nielsen and Holman 2012; Cordero-Molina et al. 2024). While it has been proposed that transgenerational immune priming in males might represent a ‘low-cost’ form of parental care that does not require substantial reproductive effort (Jokela 2010), the links between paternal transgenerational immune priming and reproductive investment remain poorly understood.

To summarize, our findings provide evidence that transgenerational immune priming in *T. molitor* exhibits heritable variation and is associated with important maternal life-history traits. The observed associations between transgenerational immune priming, fecundity, and body size align with predictions from the pace-of-life syndrome framework, and, to a lesser extent, with those of the terminal investment hypothesis, suggesting that immune priming may be integrated into broader patterns of reproductive allocation. These insights underscore the need to consider transgenerational immune priming not merely as an immune phenomenon, but as an important component of adaptive reproductive allocation shaped by selection across ecological and evolutionary contexts.

## Data archiving

Data are provided in the Supplementary Information.

## Supplementary information

Dataset
