# Supplementary material for: Genetic variation in transgenerational immune priming and its association with fecundity and body mass in the mealworm beetle Tenebrio molitor
Source: Heredity (Edinb). 2026 May 28;135(6):436–44. doi: 10.1038/s41437-026-00850-9 (PMC13354776; doi:10.1038/s41437-026-00850-9)
Supplement: Supplementary file 1 — Dataset [file 41437_2026_850_MOESM1_ESM.pdf]

Data set

Female.ID:	ID of the female individuals
Line.ID:	Inbred line ID and the stock
Female.mass:	female body mass in mg
Egg.laid:	number of eggs laid between day 2 and day 8 post-challenge
Egg.tested:	number of eggs tested for antibacterial activity
Egg.protected:	number of eggs found protected
Egg.ABA:	mean antibacterial activity of the protected eggs (mean size of the inhibition zone in cm)
Prop.protected.eggs:	proportion of protected eggs (Egg.protected/Egg.tested)

Female.ID	Line.ID	Female.mass	Egg.laid	Egg.tested	Egg.protected	Egg.ABA	Prop.protected.eggs	Survival
1	I	76.8	28	18	15	0.64	0.83	28
5	I	76	32	21	15	0.61	0.71	14
6	I	84.1	15	15	1	0.5	0.07	25
8	I	104	40	36	18	0.68	0.5	25
10	I	111.1	27	6	3	0.99	0.5	20
12	I	127.8	66	54	45	0.7	0.83	21
13	I	89.2	13	10	5	1.12	0.5	21
16	I	98.8	53	26	20	0.73	0.77	28
21	I	108	19	9	9	1.02	1	21
23	I	98.6	7	6	3	1.33	0.5	21
33	I	98.1	43	3	2	1.08	0.67	36
35	I	94.5	37	19	16	0.87	0.84	22
36	I	115.3	61	52	52	0.85	1	15
40	I	70	29	24	23	0.76	0.96	21
41	I	91.9	43	27	26	0.94	0.96	21
43	I	75.6	5	4	4	0.86	1	21
45	I	84.4	14	9	9	0.75	1	22
2	H	91.5	12	7	0		0	16
5	H	96	9	8	0		0	18
6	H	77.8	40	21	16	0.99	0.76	20
7	H	85.8	49	39	34	1.04	0.87	20
12	H	85	14	10	10	1.04	1	21
13	H	62.2	25	16	15	1.02	0.94	28
14	H	76.3	7	5	3	1.73	0.6	21
15	H	118.8	54	42	34	0.96	0.81	21
18	H	136.7	47	10	2	0.78	0.2	14
19	H	125	6	6	2	0.68	0.33	14
24	H	119.9	21	7	7	0.81	1	22
29	H	86.4	59	38	38	0.81	1	29
33	H	102.2	24	11	10	1.03	0.91	21
35	H	90.8	8	6	6	1	1	14
36	H	99.9	6	5	5	0.71	1	21
37	H	93.3	35	23	22	0.87	0.96	21
39	H	89.8	35	25	25	0.84	1	21
1	A	130	28	20	12	0.69	0.6	16
2	A	111.2	5	4	0		0	16
3	A	104.2	47	24	14	0.7	0.58	16
6	A	107.3	11	8	0		0	16
8	A	132.5	7	6	0		0	28
9	A	119.9	24	13	7	0.59	0.52	28
10	A	124.1	13	3	0		0	14
11	A	102.8	11	2	0		0	14
16	A	118.9	8	7	1	0.45	0.14	8
17	A	119.6	9	3	0		0	32
18	A	124.8	12	8	0		0	25
19	A	137.9	16	6	3	0.54	0.5	39
20	A	115	3	2	0		0	25
21	A	112.9	24	12	0		0	25
22	A	93.7	6	2	0		0	25
24	A	133.5	11	9	0		0	18
25	A	80.4	15	5	0		0	109
1	G	107.3	22	16	0		0	25
5	G	105.7	13	11	4	1.31	0.36	39
6	G	152.1	27	8	6	0.96	0.75	16
7	G	121.3	20	12	2	0.73	0.17	16
9	G	135.1	2	2	0		0	16
11	G	106.8	30	21	15	0.94	0.71	32
13	G	96.8	9	2	0		0	18

Female.ID	Line.ID	Female.mass	Egg.laid	Egg.tested	Egg.protected	Egg.ABA	Prop.protected.eggs	Survival
14	G	107	31	22	19	1.08	0.86	25
15	G	88.2	7	4	2	0.54	0.5	18
16	G	97.1	9	7	0		0	25
17	G	97.7	19	11	11	0.63	1	18
18	G	114	16	12	2	0.75	0.17	18
19	G	116.4	30	8	7	0.65	0.88	38
20	G	104.2	21	8	8	0.8	1	46
21	G	109.7	20	12	10	0.7	0.83	46
22	G	111.6	17	10	10	0.8	1	53
25	G	102.3	24	14	12	0.64	0.86	46
2	C	97.2	4	4	0		0	16
8	C	102.4	10	6	4	0.8	0.67	28
10	C	111.2	5	3	1	0.7	0.33	32
19	C	113.2	6	5	2	0.78	0.4	25
22	C	107.9	5	2	1	0.65	0.5	39
25	C	100.7	14	12	4	0.7	0.33	25
26	C	80.6	19	17	7	0.65	0.41	18
27	C	100.3	5	4	0		0	18
28	C	94.4	5	5	5	0.57	1	18
29	C	89.2	14	10	3	1.03	0.3	18
31	C	89.7	8	7	0		0	18
32	C	125.3	8	7	1	1.2	0.14	32
36	C	105.8	6	5	2	0.55	0.4	18
38	C	112.3	3	3	0		0	18
42	C	110.2	9	8	0		0	18
43	C	138.2	10	6	0		0	18
51	C	130.8	14	10	3	0.97	0.3	18
2	J	105.4	76	52	48	0.75	0.92	37
3	J	96	7	2	1	0.45	0.5	11
4	J	112.4	25	16	8	0.68	0.5	28
6	J	99.3	9	6	2	0.68	0.33	28
7	J	87.2	14	8	6	1.12	0.75	14
8	J	82.2	14	4	2	0.38	0.5	14
10	J	94.9	6	4	4	0.56	1	18
11	J	90.7	30	19	18	0.64	0.95	18
12	J	85	21	15	11	0.7	0.73	46
16	J	101.3	23	20	19	0.83	0.95	36
18	J	92.2	6	3	3	0.52	1	36
19	J	73.1	9	2	2	0.96	1	21
20	J	97.5	17	4	4	0.58	1	29
21	J	75.9	13	3	2	0.8	0.67	29
22	J	86.8	25	16	16	1.08	1	36
23	J	84.8	33	24	24	1.14	1	36
24	J	93.6	26	20	19	0.92	0.95	21
1	D	106.5	11	10	0		0	16
2	D	92.4	11	6	1	0.9	0.17	16
3	D	84.7	19	13	0		0	16
5	D	85	31	24	15	0.69	0.63	37
6	D	82.8	20	18	9	0.53	0.5	16
7	D	101.4	33	19	1	1.2	0.05	16
8	D	99.8	3	3	1	1.05	0.33	16
9	D	90.3	36	24	11	0.79	0.46	16
10	D	95.7	26	14	11	1.06	0.79	16
12	D	88.7	31	19	5	0.55	0.26	16
14	D	62.7	14	10	7	0.79	0.7	16
15	D	116.6	48	18	6	0.59	0.33	16
16	D	74.2	21	18	9	0.8	0.5	16
18	D	94.6	40	31	26	1.09	0.84	16
19	D	77	41	26	0		0	23
20	D	94.3	33	26	0		0	15
24	D	90.3	44	28	25	0.89	0.89	46
1	B	85.8	25	22	3	0.48	0.14	32
3	B	129.8	69	37	33	0.73	0.89	18
4	B	123.8	30	26	12	0.66	0.46	32
7	B	148.5	24	13	0		0	14
8	B	130.8	3	3	0		0	14
10	B	117.1	55	43	2	0.44	0.05	18
11	B	148.5	45	33	14	0.79	0.42	18
13	B	120.3	34	25	6	0.56	0.24	18
15	B	103.6	13	11	0		0	18

Female.ID	Line.ID	Female.mass	Egg.laid	Egg.tested	Egg.protected	Egg.ABA	Prop.protected.eggs	Survival
16	B	121.9	33	27	9	0.66	0.33	32
17	B	117.1	11	6	1	0.6	0.17	18
18	B	119.1	59	38	12	0.75	0.32	18
19	B	138.1	7	3	0		0	18
20	B	139.7	11	8	0		0	18
21	B	151	40	34	1	0.65	0.03	32
22	B	154.7	35	22	5	1.09	0.23	52
23	B	164.1	41	27	1	1.4	0.04	25
1	E	69.9	5	4	3	0.5	0.75	32
2	E	62.4	8	4	3	0.45	0.75	49
3	E	71.4	12	10	1	0.4	0.1	39
4	E	72.9	19	13	8	0.83	0.62	25
5	E	108.8	19	6	3	0.6	0.5	23
6	E	108.1	3	3	2	0.5	0.67	23
8	E	89.2	9	5	0		0	23
9	E	90.3	19	11	0		0	37
12	E	92.5	22	17	13	0.91	0.76	23
16	E	88.8	20	9	8	1.01	0.89	23
18	E	94.9	4	3	3	0.85	1	16
20	E	72.7	11	7	0		0	23
21	E	96.7	8	5	2	0.8	0.4	28
22	E	91.4	24	14	6	0.69	0.43	32
24	E	119.2	42	17	6	0.6	0.35	32
26	E	119.4	30	18	1	0.7	0.06	81
27	E	86.1	19	11	6	0.63	0.55	67
10	F	107	10	7	6	0.94	0.86	60
12	F	113	12	8	0		0	60
14	F	99.2	14	9	6	0.96	0.67	46
19	F	91.5	6	6	5	0.89	0.83	32
20	F	134.6	38	31	1	0.45	0.03	18
27	F	128.8	36	24	4	0.95	0.17	27
28	F	128.8	6	6	1	0.8	0.17	20
29	F	140	21	19	9	0.7	0.47	20
32	F	141.1	47	35	15	0.72	0.43	13
33	F	108.9	38	36	25	0.82	0.69	13
35	F	106.9	44	11	9	0.14	0.82	21
36	F	85.4	24	13	8	0.1	0.62	21
38	F	135	19	17	6	0.73	0.35	29
39	F	155	27	23	12	0.98	0.52	21
41	F	111	13	11	9	1.09	0.82	28
44	F	118.5	38	31	26	0.88	0.84	21
46	F	123.1	15	14	6	0.74	0.43	21
1	Stock	110.1	51	46	1	0.9	0.02	25
2	Stock	100.2	87	50	1	1.05	0.02	67
3	Stock	101.8	54	42	18	0.64	0.43	46
4	Stock	116.7	58	36	27	0.69	0.75	46
8	Stock	124.6	59	35	1	1	0.03	46
9	Stock	109.9	58	50	0		0	32
10	Stock	136.4	72	63	52	0.9	0.83	53
11	Stock	109.9	37	25	21	1.03	0.84	60
14	Stock	109.5	48	27	12	0.64	0.44	25
22	Stock	85.9	58	37	30	0.76	0.81	53
23	Stock	119.3	52	38	3	0.28	0.08	60
24	Stock	99.8	45	33	7	0.56	0.21	46
25	Stock	107.3	79	61	3	0.53	0.05	32
26	Stock	88.9	57	30	19	0.71	0.63	39
27	Stock	97.2	68	38	5	0.51	0.13	60
29	Stock	98	52	20	0		0	60
30	Stock	97.9	52	37	26	0.54	0.7	53
